# Preparation and Evaluation of a Self-Nanoemulsifying Drug Delivery System Loaded with Heparin Phospholipid Complex

**DOI:** 10.3390/ijms22084077

**Published:** 2021-04-15

**Authors:** Xiao-Lei Qiu, Zi-Rui Fan, Yang-Yang Liu, Ding-Fu Wang, Shi-Xin Wang, Chun-Xia Li

**Affiliations:** 1 Key Laboratory of Marine Drugs of Ministry of Education, Shandong Provincial Key Laboratory of Glycoscience and Glycoengineering, School of Medicine and Pharmacy, Ocean University of China, Qingdao 266003, China; chouxiaolei@stu.ouc.edu.cn (X.-L.Q.); 21190811084@stu.ouc.edu.cn (Z.-R.F.); 21180831070@stu.ouc.edu.cn (Y.-Y.L.); wangdingfu@stu.ouc.edu.cn (D.-F.W.); wsx8516@ouc.edu.cn (S.-X.W.); 2 Marine Biomedical Research Institute of Qingdao, Qingdao 266071, China; 3 Laboratory for Marine Drugs and Bioproducts, Pilot National Laboratory for Marine Science and Technology (Qingdao), Qingdao 266237, China

**Keywords:** heparin, heparin–phospholipid complex, self-nanoemulsifying drug delivery system, oral absorption

## Abstract

A self-nanoemulsifying drug delivery system (SNEDDS) was developed to enhance the absorption of heparin after oral administration, in which heparin was compounded with phospholipids to achieve better fat solubility in the form of heparin-phospholipid (HEP-Pc) complex. HEP-Pc complex was prepared using the solvent evaporation method, which increased the solubility of heparin in *n*-octanol. The successful preparation of HEP-Pc complex was confirmed by differential scanning calorimetry (DSC), Fourier-transform infrared (FT-IR) spectroscopy, NMR, and SEM. A heparin lipid microemulsion (HEP-LM) was prepared by high-pressure homogenization and characterized. HEP-LM can enhance the absorption of heparin after oral administration, significantly prolong activated partial thromboplastin time (APTT) and thrombin time (TT) in mice, and reduce fibrinogen (FIB) content. All these outcomes indicate that HEP-LM has great potential as an oral heparin formulation.

## 1. Introduction

Unfractionated heparin (UFH) is a natural and widely used anticoagulant drug with good antithrombotic effects [1,2,3]. Its main route of administration is intravenous injection or subcutaneous injection, which limits the long-term use of heparin. Effective oral heparin preparations can greatly increase patient compliance. However, due to the high molecular weight, high negative charge, and susceptible enzymatic degradation of heparin, direct oral administration of heparin is ineffective. In order to overcome the poor bioavailability of oral heparin, several research groups have tried various drug delivery systems, such as liposomes, complexes of heparin and hydrophobic organic bases, enteric coatings, and aerosols [4,5,6]. At the same time, some attempts have been made to evaluate the absorption enhancement effect of ethylenediaminetetraacetic acid (EDTA), acid buffer, *N*-[8-(-(hydroxybenzoyl) amino] sodium octanoate (SNAC), or sulfated surfactant on heparin in the gastrointestinal tract [7,8,9]. However, no oral heparin preparation has entered the market at present.

In recent years, nanoscaled drug delivery has attracted great interest due to its advantages of producing good pharmacological effectiveness, as well as less toxicity [10]. Lipid microemulsions seem to be a good carrier for drug delivery with some advantages, such as thermodynamic stability (long life), easy formation (zero interfacial tension), low viscosity, high surface area (high solubility), and small droplet size [11,12,13]. The self-emulsifying drug delivery system, also known as the self-nanoemulsifying drug delivery system (SNEDDS), is a new isotropic system composed of an oil phase, a surfactant, and a co-surfactant [14,15]. SNEDDS improves drug delivery by improving the transport of drugs with poor permeability, weakening the activity of intestinal excretion transporters, preventing the degradation of drugs in the physiological environment, and promoting the absorption of drugs through the intestinal lymphatic pathway [15]. Due to these advantages, SNEDDS was applied to improve the oral absorption of hydrophobic drugs such as nimodipine [16], talinolol [17], and paclitaxel [18]. However, the application of hydrophilic drugs in SNEDDS was limited. For oil-in-water (O/W) lipid microemulsions, water-soluble drugs may be lost to the environmental aqueous medium in vivo. For water-in-oil (W/O) lipid microemulsions, when the preparation is diluted with a relatively large amount of gastrointestinal fluid, phase inversion may occur in the body and cause water-soluble drug leakage. For water-soluble drugs, solid dispersion technology is often used, such as β-lactamase. However, studies have shown that nearly 50% of the β-lactamase loaded in SNEDDS leaks out of the oil phase when mixed with phosphate-buffered saline [19]. In order to increase the application of water-soluble drugs, there several studies were carried out to develop SNEDDS of hydrophilic drugs through a phospholipid complex strategy, in which a phospholipid complex was formulated to enhance the fat solubility of water-soluble drugs and promote their incorporation into SNEDDS. The oral absorption of matrine [20] and insulin [21] was significantly improved by this special formulation. So far, these works have mainly focused on small-molecule drugs and plant extracts, such as silybin, curcumin, and naringin [22,23,24]. Few studies have systematically studied the preparation of SNEDDS for sulfated polysaccharides such as heparin.

In this study, heparin was formulated into a new drug–phospholipid complex using the solvent volatilization method for the first time to enhance its lipophilicity and oral bioavailability. We use the method of mixed solvents to solve the problem of low reaction rate caused by the poor solubility of heparin in organic solvents. The heparin self-nanoemulsifying drug delivery system was successfully prepared on the basis of a heparin–phospholipid (HEP–Pc) complex, which increased the oral absorption of heparin, and it provides a feasible strategy for the clinical application of oral heparin.

## 2. Results

### 2.1. HEP–Pc Complex Preparation and Its Liposolubility

The heparin–phospholipid (HEP–Pc) complex was prepared using the solvent evaporation method [25]. After the optimization of preparation conditions, heparin and phospholipid were used to prepare the HEP–Pc complex at a mass ratio of 1:6.6. The aqueous solution of heparin was mixed with phospholipid (soy lecithin) in dichloromethane (DCM) to prepare the HEP–Pc complex. After completion of the complex reaction, the solvent was removed, and DCM was added to redissolve the HEP–Pc complex. The uncomplexed heparin was insoluble in DCM, allowing its collection by centrifugation. The association efficiency of heparin in the HEP–Pc complex was 99.62% ± 0.25% (*n* = 3). Heparin is water-soluble and difficult to dissolve in the organic solvent of DCM. During the preparation process, it was found that the solubility of heparin in DCM could not be improved by simple physical mixing with phospholipids. On the contrary, the HEP–Pc complex was easily dissolved in DCM.

**Figure 1:**
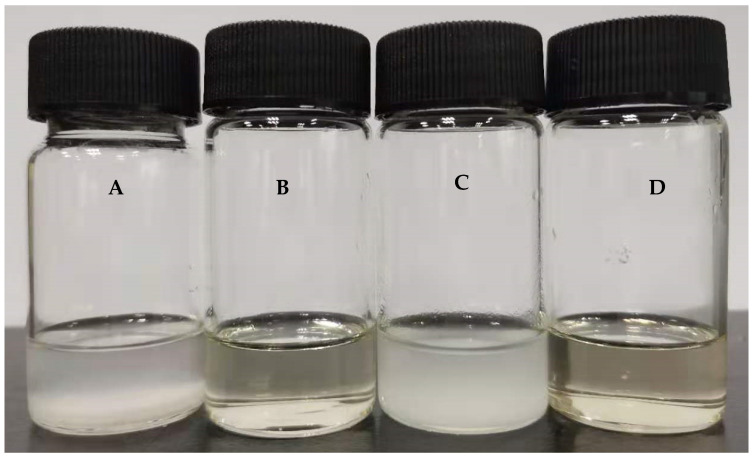
The solubility of heparin (**A**), phospholipids (**B**), physical mixture of heparin and phospholipids (**C**), and heparin–phospholipid (HEP–Pc) complex (**D**) in *n*-octanol.

Heparin is a sulfate polysaccharide drug, and it has high water solubility, but poor fat solubility and poor cell membrane permeability, leading to its inability to be absorbed orally. *n*-Octanol is usually used as a solvent to investigate the liposolubility of drugs in oil–water distributions. The solubility of the HEP–Pc complex in *n*-octanol was evaluated. After 24 h of shaking at 37 °C, the solubility of heparin, phospholipids, their physical mixture, and the HEP–Pc complex in *n*-octanol was as shown in Figure 1. The drug concentration added to *n*-octanol was 5 mg/mL (equivalent to heparin). Figure 1A,C showed that heparin and the physical mixture of heparin and phospholipids could not be dissolved in *n*-octanol, and the solution was in a turbid state, indicating that simply physically mixing heparin and phospholipids cannot increase the liposolubility of heparin. Figure 1D shows that the HEP–Pc complex was well dissolved in *n*-octanol, and the solution was clear, indicating that the phospholipid complex technology increased the fat solubility of heparin and was beneficial for improving the oral absorption of heparin.

### 2.2. Characterization of the HEP–Pc Complex

#### 2.2.1. Fourier-Transform Infrared (FT-IR) Analysis

**Figure 2:**
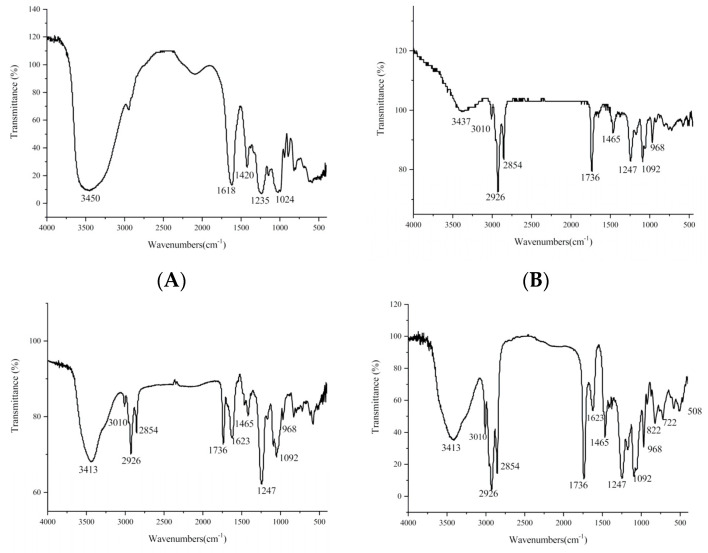
Fourier-transform infrared (FT-IR) spectra of (**A**) heparin, (**B**) phospholipids, (**C**) physical mixture of heparin and phospholipids, and (**D**) HEP–Pc complex.

The interaction between heparin and phospholipids was studied using Fourier-transform infrared spectroscopy (FT-IR) (Figure 2). Figure 2A,B show the IR spectra of heparin and phospholipid, respectively. Figure 2C shows that the spectrum of the physical mixture was a superposition of the peaks of heparin and phospholipid. There was no obvious change in the intensity and displacement of each peak, indicating that there was no interaction between heparin and phospholipid. However, the IR spectrum of the HEP–Pc complex (Figure 2D) had obvious change. The hydroxyl stretching vibration peak of heparin shifted from 3450 cm^−1^ to 3413 cm^−1^, and the νas*_COO_^−^* peak of heparin at 1623 cm^−1^ and the ν*_P_*_=*O*_ peak of phospholipid at 1247 cm^−1^ were significantly weakened, but the hydrocarbon stretching vibration of the nonpolar end of the phospholipid at 2925 cm^−1^ and 2853 cm^−1^ was obviously enhanced. It was speculated that the carboxyl and hydroxyl groups in heparin interacted with the hydroxyl and phosphorus–oxygen groups of the phospholipid to form hydrogen bonds, which led to a significant decrease in peak intensity, and the polar groups related to heparin were wrapped inside, whereas the long CH chain was outside, which enhanced the vibration absorption of the CH bond [26]. However, no new peaks appeared, indicating that no new chemical bonds were formed between phospholipids and polysaccharides. The IR spectrum of the heparin–phospholipid complex showed that a complex different from the original monomer was formed between heparin and phospholipid, which proved the formation of the HEP–Pc complex.

#### 2.2.2. Nuclear Magnetic Resonance (NMR) Analysis

**Figure 3:**
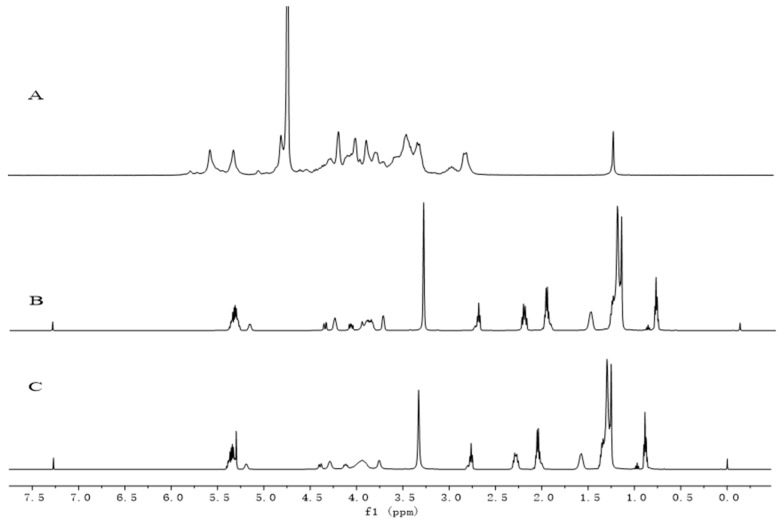
^1^H-NMR of (**A**) heparin (in D_2_O), (**B**) Pc (in CDCl_3_), and (**C**) the HEP–Pc complex (in CDCl_3_).

The ^1^H-NMR spectra of heparin, phospholipids, and the HEP–Pc complex are shown in Figure 3. The ^1^H-NMR spectrum of the HEP–Pc complex (Figure 3C) mainly displayed the signal peaks of phospholipid (Figure 3B), while the peaks of heparin (Figure 3A) disappeared. A possible reason is that the HEP–Pc complex mainly existed in the form of reverse micelles in deuterated chloroform. In reverse micelles, the polar end of the drug molecule was inward, while the nonpolar end was outward. Multiple HEP–Pc complex molecules were arranged in an orderly manner to form a closely arranged clump. This structure had a shielding effect on the drug molecules of heparin, such that the signals of heparin were not clearly displayed in the ^1^H-NMR of the HEP–Pc complex. To a certain extent, it also affected the polar end of phospholipid molecules, and its corresponding signals became wider and weaker (3.5–4.5 ppm), while the nonpolar end of phospholipid molecules could rotate freely, such that its corresponding signals did not change [27].

#### 2.2.3. Differential Scanning Calorimetry (DSC) Analysis

Differential scanning calorimetry (DSC) is a reliable method to explore the possible interactions between a drug and excipients. In DSC, an interaction can be concluded by elimination of an endothermal peak, appearance of a new peak, changes in peak shape and its onset, peak temperature/melting point, and relative peak area or enthalpy [20].

**Figure 4:**
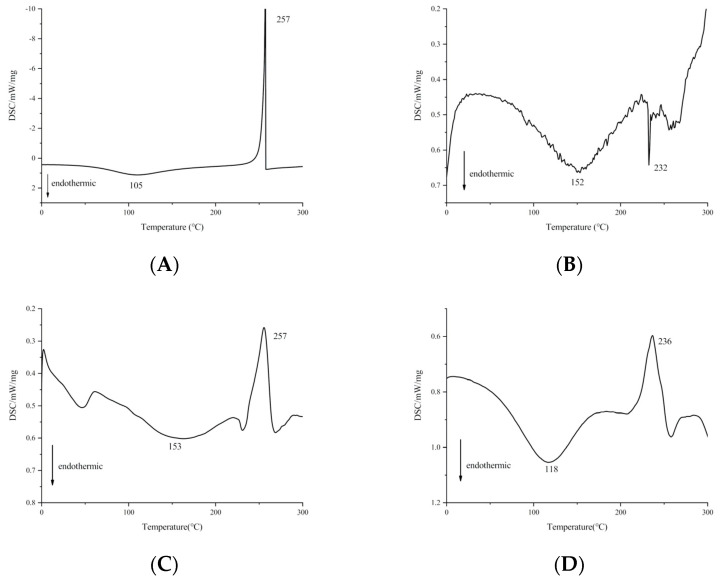
Differential scanning calorimetry (DSC) thermograms of (**A**) heparin, (**B**) phospholipids, (**C**) physical mixture of heparin and phospholipids, and (**D**) the HEP–Pc complex.

The results of DSC analysis are shown in Figure 4. Heparin had two different peaks (Figure 4A). The first was the endothermal peak at 105 °C, which might be due to the phase change of heparin from solid to liquid. The second was the exothermic peak at 257 °C, which was caused by the decomposition of heparin by heat. Figure 4B shows that the phospholipids had two endothermal peaks. The first endothermic peak appeared at 152 °C, indicating the thermal movement of the polar part of the phospholipid molecule. The second endothermic peak appearing at 232 °C was attributed to the transition from the gel state to the liquid crystal state, which was due to the melting of the hydrocarbon chain in the phospholipid and the change of isomers or crystals [28,29]. The physical mixture of heparin and phospholipids showed an endothermic peak at 153 °C and an exothermic peak at 257 °C (Figure 4C), which were the superposition of heparin and phospholipids; no new peaks appeared. For the HEP–Pc complex, Figure 4D shows that the endothermal peaks of heparin and phospholipid disappeared, and a new phase transition temperature (118 °C) was formed. A possible reason is that, after the combination of heparin and the polar part of the phospholipid molecule, the hydrocarbon chains in the phospholipids could freely rotate and wrap the polar part of the phospholipid molecule, which reduced the sequential energy between the phospholipid aliphatic hydrocarbon chain and led to the disappearance of the endothermal peak of phospholipids, whereby the phase transition temperature decreased [15]. These phenomena also confirmed the formation of the HEP–Pc complex.

#### 2.2.4. SEM Analysis

**Figure 5:**
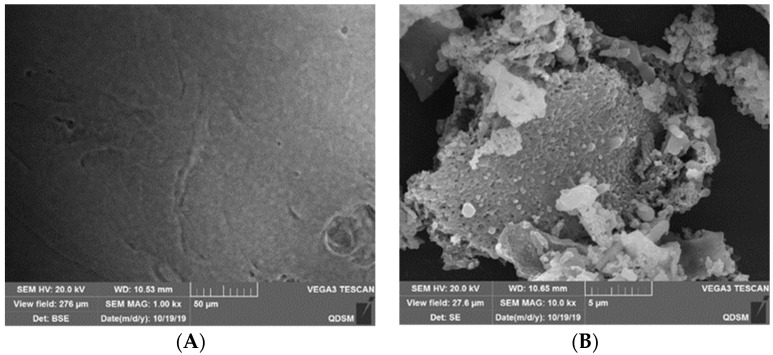

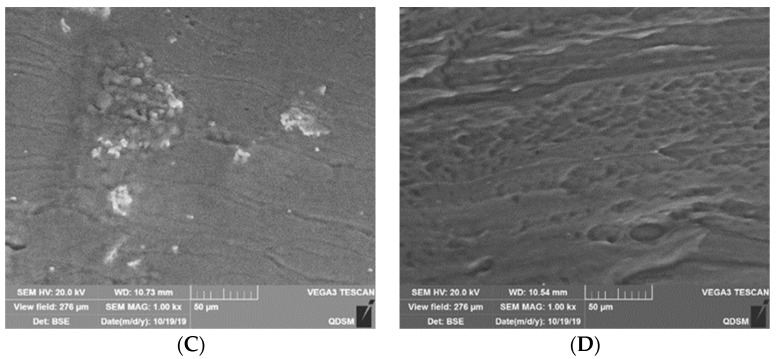
Scanning electron microscopy (SEM) of (**A**) phospholipids, (**B**) heparin, (**C**) physical mixture of heparin and phospholipids, and (**D**) the HEP–Pc complex.

Scanning electron microscopy (SEM) is used to extract various physical information through the interaction between a high-energy electron beam and matter, thus obtaining the three-dimensional shape of the matter surface. Figure 5A shows that the phospholipids were in an amorphous state without particulate matter. Unlike phospholipids, heparin was irregularly arranged and granular (Figure 5B). Figure 5C shows that the granular shape of heparin was clearly seen in the physical mixture of heparin and phospholipid. Heparin and phospholipid were simply mixed, and there was no morphological change and no change in the surface state of the substance. Figure 5D shows that the surface of the HEP–Pc complex was smooth, and the particle state of heparin molecules disappeared, indicating that heparin molecules were evenly dispersed in the phospholipid matrix and interacted with phospholipids to form a phospholipid complex, which was completely different from physical mixtures.

The solvent volatilization method was used to prepare the heparin–phospholipid complex. IR and ^1^H-NMR showed that the HEP–Pc complex had no new absorption peaks. DSC showed that the absorption peaks of heparin and phospholipids basically disappeared, and new absorption peaks appeared. SEM showed that the HEP–Pc complex was a homogeneous substance. All the above analyses confirmed the successful preparation of the heparin–phospholipid complex.

### 2.3. Preparation and Characterization of HEP-LM

**Figure 6:**
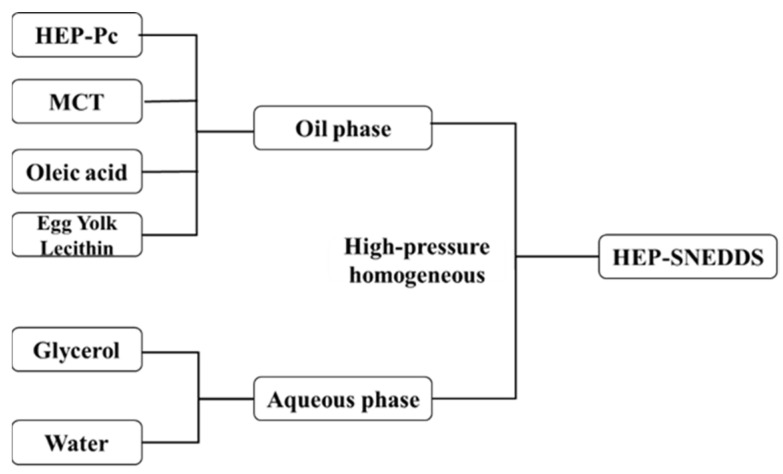
The scheme of the self-nanoemulsifying drug delivery system of heparin (HEP-SNEDDS) preparation.

In order to improve the oral absorption of heparin, the heparin–phospholipid complex (HEP–Pc complex) was further prepared into a heparin lipid microemulsion (HEP-LM), which formed the self-nanoemulsifying drug delivery system of heparin (HEP-SNEDDS). The preparation procedure of HEP-SNEDDS is shown in Figure 6. The optimized formulation of HEP-SNEDDS is listed in Table 1.

**Figure 7:**
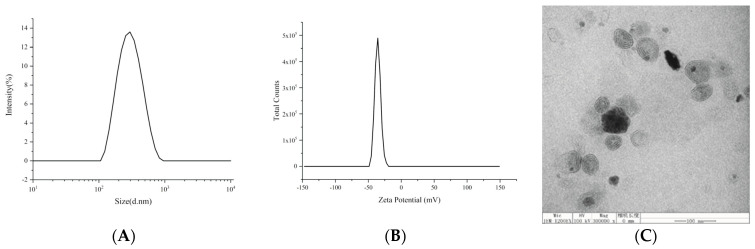
Characterization of heparin lipid microemulsion (HEP-LM): (**A**) particle size distribution; (**B**) zeta potential; (**C**) TEM images (scale bar represents 100 nm).

Medium-chain triglyceride (MCT) was selected as the oil because the HEP–Pc complex had better solubility in it than long-chain oils, and egg yolk lecithin was added as an emulsifier in the oil phase to achieve better emulsification. Oleic acid (OA) and glycerol were used as a stabilizer and an iso-osmotic adjusting agent, respectively. The oil phase was added to the water phase, and the heparin lipid emulsion (HEP-LM) was obtained through high-pressure homogenization. The particle size of HEP-LM was ~180 nm, and the polydispersity index (PDI) was less than 0.2 (Table 2, Figure 7A,B), indicating that the heparin emulsions were uniform and had a narrow particle size distribution. The morphology of the HEP-LM in Figure 7C shows that the formulation was well dispersed, and the emulsion particles had a spherical shape with clear multiple bilayers of phospholipids. Heparin was dissolved in the oil phase in the form of a phospholipid complex and was simultaneously encapsulated by lecithin, which improved the encapsulation efficiency (EE%) of heparin. The EE% of HEP-LM was 95.7%. HEP-LM had low surface tension and easily passed through the hydration layer of gastrointestinal wall, such that the emulsion drops could directly contact the gastrointestinal epithelial cells, making heparin more easily absorbed by oral administration.

### 2.4. In Vivo Anticoagulation of HEP–Pc Complex and HEP-LM in Mice

**Figure 8:**
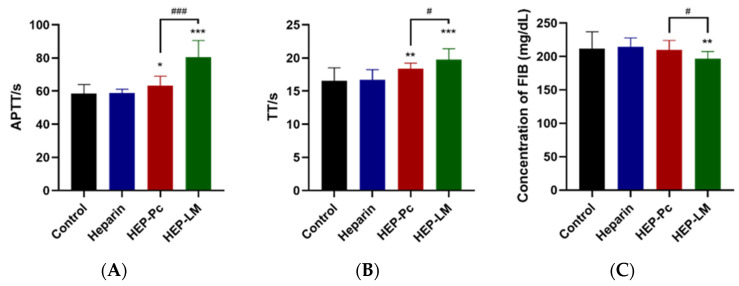
Anticoagulation of the HEP–Pc complex and HEP-LM oral administration in mice on (**A**) activated partial thromboplastin time (APTT), (**B**) thrombin time (TT), and (**C**) fibronectin (FIB). Values are expressed as the mean ± SD. * *p* < 0.05, ** *p* < 0.01, and *** *p* < 0.001 compared with oral heparin; # *p* ˂ 0.05 and ### *p* ˂ 0.001 compared with HEP–Pc complex.

Heparin is the most widely used anticoagulant drug with good antithrombotic function. Figure 8 shows the activated partial thromboplastin time (APTT), thrombin time (TT), and fibronectin (FIB) of the heparin, HEP–Pc complex, and HEP-LM oral administration in vivo. The heparin oral group had no significant change compared with the control group, indicating that oral heparin cannot exert its anticoagulant and antithrombotic effects. Compared with the oral heparin group, the HEP–Pc complex could significantly prolong APTT and TT (*p* < 0.01, *p* < 0.05), indicating that the phospholipid complex technology can improve the oral absorption of heparin. Compared with the oral heparin group, HEP-LM could significantly prolong APTT and TT (*p* < 0.001) and reduce the content of FIB (*p* < 0.01), with a more significant effect than the HEP–Pc complex, indicating that the lipid self-emulsification technology can further improve the oral absorption of heparin.

Phospholipids, which maintain cell membrane fluidity and cell integrity, are an important part of the membrane phospholipid bilayer. Phospholipid complexes can increase the biocompatibility of drugs. The main reason for the phospholipid complex improving the oral absorption of heparin was the increase in its fat solubility. The phospholipid complex/self-emulsifying drug delivery system combined heparin into a phospholipid complex, which increased the contact area between the drug and gastrointestinal tract, increased the absorption of the drug through intestinal lymph, and avoided the first-pass effect of the drug through the liver. Therefore, the oral absorption of heparin was significantly improved through the combination of the phospholipid complex and self-emulsification technology.

## 3. Discussion

An HEP–Pc complex was prepared using a solvent evaporation method. The choice of solvents was the key point for the preparation of the complex. It is well known that water-soluble sulfated polysaccharides, such as heparin, cannot be directly dissolved in organic solvents. In our study, the HEP–Pc complex was successfully prepared by using a mixed solvent of dichloromethane and water to increase the solubility of heparin. At the same time, the polar end of the phospholipid faced outward due to the presence of water, which increased the probability of binding with heparin.

From the characterization results of the complex, infrared spectroscopy showed that the HEP–Pc complex did not form new chemical bonds, but the polar heads of heparin and phospholipid molecules combined in the form of hydrogen bonds or van der Waals force to form the heparin–phospholipid complex. The ^1^H-NMR spectrum showed that the signal of heparin in the HEP–Pc complex disappeared, the signal of the polar end of the phospholipid was weakened, and the signal of the aliphatic chain end remained constant, indicating the interaction between heparin and the polar end of the phospholipid molecule to form the HEP–Pc complex. This structure was easily soluble in the oil phase because the fat-soluble tails of the phospholipids were outward. DSC showed that, after the formation of the phospholipid complex, the characteristic endothermic peaks of heparin and phospholipid basically disappeared, and the phase transition temperature of the phospholipid complex was lower than the free phospholipid, indicating the formation of the drug-and-phospholipid complex. SEM showed that the physical mixture of heparin and phospholipids presented heparin granular material, while a parallel granular material was seen in the HEP–Pc complex, fully indicating that heparin molecules were dispersed in the phospholipid matrix and interacted with the phospholipids to form a phospholipid complex. 

After heparin formed a phospholipid complex, its fat solubility was greatly improved; then, an HEP-LM with an encapsulation efficiency as high as 95.7% was successfully prepared. In vivo anticoagulation experiments showed that the anticoagulation effect of HEP-LM was much stronger than oral heparin and the HEP–Pc complex, suggesting that the bioavailability of the heparin phospholipid complex was further improved with the lipid microemulsion preparation.

The HEP–Pc complex formed via this method acts as a bridge connecting conventional and novel delivery systems because of the significant improvement in its lipophilicity. It can be dissolved in an oil phase or an organic solvent to prepare an O/W emulsion, self-emulsion, micelle, or nanoparticulate carrier. These drug delivery carriers make the noninvasive delivery of heparin a promising alternative to the route of subcutaneous injection.

## 4. Materials and Methods

### 4.1. Materials and Animals

Heparin (molecular weight (Mw) = 14 kDa, 185 USP/mg) was purchased from Shanghai Aladdin Biochemical Technology Co., Ltd. (Shanghai, China). Soy lecithin was purchased from Shanghai Taiwei Pharmaceutical Co., Ltd. (Shanghai, China). Azure A was purchased from Beijing Soleibao Technology Co., Ltd. (Beijing, China). Egg yolk lecithin was purchased from AVT Pharmaceutical Technology Co., Ltd. (Shanghai, China). Dichloromethane and other chemical reagents were purchased from China Pharmaceutical Group Co., Ltd. (Beijing, China). The activated partial thromboplastin time kit (APTT), thrombin time kit (TT), and fibrinogen determination kit (FIB) were purchased from Shanghai Sun Biotechnology Co., Ltd. (Shanghai, China). All other chemicals were of analytical or chromatography grade. 

SPF-grade Kunming male mice (4–6 weeks; body weight 20–26 g) were purchased from the Jinan Peng Yue Laboratory Animal Breeding Center (Jinan, Shandong Province, China). All animal experiment protocols were approved by the Animal Care and Use Committee of the Ocean University of China and conducted in accordance with the National Institutes of Health (NIH) Guidelines for the Care and Use of Laboratory Animal. All mice were housed in an environmentally controlled room at 22 ± 2.0 °C, 50% ± 5% humidity, with a 12 h light/dark cycle, and they were given water ad libitum.

### 4.2. Preparation of Heparin–Phospholipid Complex

The heparin–phospholipid (HEP–Pc) complex was prepared using a solvent evaporation method [25]. Briefly, heparin (0.2 g) dissolved in 10 mL of water and phospholipid (1.32 g) dissolved in 10 mL of dichloromethane (DCM) were mixed together in a round-bottom flask and stirred for 6 h in a 40 °C water bath. Then, the solvent was removed by vacuum rotary evaporation and dried in a vacuum-drying oven at 60 °C for 2 h. The residue was redissolved in dichloromethane (5 mL) and centrifuged to isolate uncomplexed heparin precipitate. The supernatant solvent was removed by vacuum rotary evaporation to obtain the HEP–Pc complex as pale yellow waxy solid.

The HEP–Pc complex and phospholipids were easily dissolved in dichloromethane. Uncomplexed heparin (free heparin) was insoluble in dichloromethane and collected by centrifugation. The association efficiency was calculated using Equation (1).

Association efficiency (%) = (*W*_0_ − *W*_1_)/*W*_0_ × 100%, (1)

where *W*_0_ is the total mass of heparin, and *W*_1_ is the mass of uncomplexed heparin.

The liposolubility of the HEP–Pc complex was evaluated with *n*-octanol. First, 5 mL of *n*-octanol was added to the heparin (25 mg), phospholipid (165 mg), HEP–Pc complex (191 mg), physical mixture of heparin (25 mg), and phospholipid (165 mg). The solubilization of each system was evaluated after shaking at 37 °C for 24 h by visual examination. 

### 4.3. Characterization of the HEP–Pc Complex

The infrared spectra (FT-IR) of heparin, phospholipids, the HEP–Pc complex, and the physical mixture of heparin and phospholipids were recorded using an NEXUE 470 FT-IR spectrometer (Thermo Fisher Scientific, MA, USA) with a range of 4000–400 cm^−1^ and a resolution of 4 cm^−1^.

The nuclear magnetic resonance (NMR) spectra of heparin, phospholipids (soybean lecithin) and heparin phospholipid complex were recorded using a Bruker 400 MHz NMR (Bruker, Karlsruhe, Germany).

Thermograms of heparin, phospholipid, the physical mixture, and the HEP–Pc complex were recorded on a differential scanning calorimeter (DSC) (Mettler Toledo, Zurich, Switzerland) using the Mettler Stare system. Each sample (3 mg) was sealed in an aluminum crucible and heated at a rate of 10 °C·min^−1^ from 20 to 300 °C.

The morphology and surface characteristics of heparin, phospholipids, the HEP–Pc complex, and the physical mixture of heparin and phospholipids were examined using a JEOL JSM840 scanning electron microscope (SEM) (JEOL Ltd., Tokyo, Japan).

### 4.4. Preparation of HEP-LM

First, 3.1 g of the HEP–Pc complex was dissolved in DCM (5 mL); then, 2.0 g of egg yolk lecithin, 8.0 g of medium-chain triglycerides (MCTs), and 0.1 g of oleic acid were added. DCM was removed with a rotary evaporator and heated to 70 °C to obtain an oil phase. Next, 1.6 g of glycerol was dissolved in 65 mL of water and heated to 70 °C to obtain the water phase. The oil phase was slowly added dropwise to the water phase, and a coarse emulsion was obtained by high-speed shearing (FJ-200; Shanghai Specimen Model Factory; Shanghai, China) at 20,000 rpm for 5 min. The formed coarse emulsion was further circulated for 15 cycles through a high-pressure homogenizer (ATS AH-Basic; SEEKER Industries, Brampton, Canada) at 1000 bar to afford the heparin lipid emulsion (HEP-LM), and the volume was replenished to 80 mL after the cycle was finished.

### 4.5. Characterization of the HEP-LM

The size distribution, polydispersity index (PDI), and zeta potential of droplets of the heparin lipid emulsion (HEP-LM), diluted with distilled water (1:100, *w*/*w*), were measured via the dynamic light scattering method using a Zetasizer Nano ZS90 (Malvern, Worcestershire, UK). All measurements were repeated three times (*n* = 3), and their values were expressed as the mean ± standard deviation (SD).

The morphology of the HEP-LM was visualized using a transmission electron microscope (TEM) (JEM-2100F, JEOL Ltd., Tokyo, Japan).

The encapsulation efficiency (EE%) of the HEP-LM was determined using a modified Azure A colorimetric method [6]. First, 2 mL of HEP-LM (5 mg/mL) was taken and dried under reduced pressure using a diaphragm pump. The residue was added to 5 mL of dichloromethane and sonicated to fully dissolve. The solution was centrifuged at 13,500 rpm for 20 min, and the supernatant was discarded. Dichloromethane was added to wash the precipitate three times, before blow-drying with nitrogen to give free heparin. Next, 1 mL of double-distilled water was added to completely dissolve free heparin. The content of free heparin was determined according to the Azure A colorimetric method, and the encapsulation efficiency (EE%) was calculated using Equation (2).

EE% = ((weight of heparin − weight of free heparin))/(weight of heparin) × 100%.(2)

### 4.6. In Vivo Anticoagulation Study

Forty male Kunming mice were randomly divided into four groups: control group, heparin group, HEP–Pc complex group, and HEP-LM group, with 10 mice in each group. At room temperature, gavage was started after 3 days of adaptive feeding. The dosage of each group except the control group was 150 mg/kg (the dose of heparin), and the control group was given an equal volume of saline. After 7 days of continuous administration, 1 h after the last administration, blood was obtained by extirpating the eyeballs, and sodium citrate was added to mix gently (the ratio of blood to sodium citrate was 9:1). The plasma was separated by centrifugation at 4000 rpm for 15 min; then, APTT, TT, and FIB were determined according to the kit instructions.

## 5. Conclusions

In summary, we prepared an HEP–Pc complex using a solvent evaporation method, which increased the fat solubility of heparin. DSC, FT-IR, NMR, and SEM analyses confirmed the successful preparation of the HEP–Pc complex. Then, hydrophilic heparin was loaded into the self-emulsifying drug delivery system, to form a heparin lipid microemulsion (HEP-LM), through the phospholipid complex technology for the first time. HEP-LM improved the oral absorption of heparin, and it provides a new strategy for the clinical application of heparin. Our current work provides some new insights and examples for enhancing the oral bioavailability of water-soluble heparin drugs.

In the future, we will study the long-term toxicity and clinical application of the system, as well as investigate the stability of the system in gastrointestinal tract. The pharmacokinetics of the drugs should be studied by investigating the influence of dosage form and dosage on drug absorption. In the future, we will apply this technology to other polysaccharide drugs to provide technical support for the noninvasive drug delivery of polysaccharides.

## Figures and Tables

**Table 1:** Optimized formulation of HEP-SNEDDS.

Ingredients	Contents	Proportions
HEP–Pc complex	3.1 g	5 mg/mL
OA	0.1 g	0.125% (*w*/*v*)
EPC	2.0 g	2.5% (*w*/*v*)
MCT	8.0 g	10% (*w*/*v*)
Glycerol	1.6 g	2% (*w*/*v*)
Water	Added to 80 mL	-----

Abbreviations: HEP–Pc complex, heparin–phospholipid complex; OA, oleic acid; EPC, egg phosphatidylcholine; MCT, medium-chain triglyceride.

**Table 2:** Physicochemical characterization of HEP-LM (mean ± SD, *n* = 3). PDI, polydispersity index; EE%, encapsulation efficiency.

Formulations	Particle Size (nm)	Zeta Potential (mV)	PDI	EE%
HEP-LM	188.5 ± 0.5	−36.8 ± 0.5	0.1 ± 0.02	95.7 ± 0.3

## Data Availability

Data are all contained within the manuscript.
